# Solid-supported polymer–lipid hybrid membrane for bioelectrochemistry of a membrane redox enzyme†

**DOI:** 10.1039/d4lf00362d

**Published:** 2025-02-11

**Authors:** Rosa Catania, George R. Heath, Michael Rappolt, Stephen P. Muench, Paul A. Beales, Lars J. C. Jeuken

**Affiliations:** a School of Chemistry, University of Leeds Leeds LS2 9JT UK P.A.Beales@leeds.ac.uk; b Astbury Centre for Structural Molecular Biology, University of Leeds Leeds LS2 9JT UK; c School of Physics and Astronomy, University of Leeds Leeds LS2 9JT UK; d School of Food Science and Nutrition, University of Leeds Leeds LS2 9JT UK; e School of Biomedical Sciences, Faculty of Biological Sciences, University of Leeds Leeds LS2 9JT UK; f Leiden Institute of Chemistry, Leiden University PO Box 9502 2300 RA Leiden The Netherlands L.J.C.Jeuken@lic.leidenuniv.nl

## Abstract

Hybrid membranes, consisting of phospholipids and amphiphilic block polymers, offer enhanced stability compared to liposomes and greater biocompatibility than polymersomes. These qualities make them a versatile platform for a wide range of applications across various fields. In this study, we have investigated the ability of solid-supported polymer–lipid hybrid membranes (SSHM) to act as a platform for bioelectrochemistry of membrane proteins. The redox enzyme, cytochrome *bo*_*3*_ (cyt *bo*_*3*_), a terminal oxidase in *Escherichia coli*, was reconstituted into hybrid vesicles (HVs), which were subsequently tested for their ability to form SSHMs on different self-assembled monolayers (SAMs) on gold electrodes. SSHM formation was monitored with electrochemical impedance spectroscopy (EIS), quartz crystal microbalance with dissipation (QCM-D), and atomic force microscopy (AFM). SSHMs were successfully formed on gold electrodes with mixed SAMs of 6-mercapto-1-hexanol and 1-hexanethiol at a 1 : 1 ratio. The activity of cyt *bo*_*3*_ was confirmed using cyclic voltammetry (CV), with electron transfer to cyt *bo*_*3*_ mediated by a lipophilic substrate-analogue decylubiquinone (DQ). SSHMs formed with HVs-cyt *bo*_*3*_ samples, stored for more than one year before use, remain bioelectrocatalytically active, confirming our previously established longevity and stability of HV systems.

## Abstract 2

Solid-supported polymer–lipid hybrid membranes (SSHM) on gold electrodes, modified with mixed SAMs of 6-mercapto-1-hexanol and 1-hexanethiol at a 1 : 1 ratio, display oxidoreductase activity of the co-imbedded cytochrome *bo*_*3*_.

## Graphical abstract

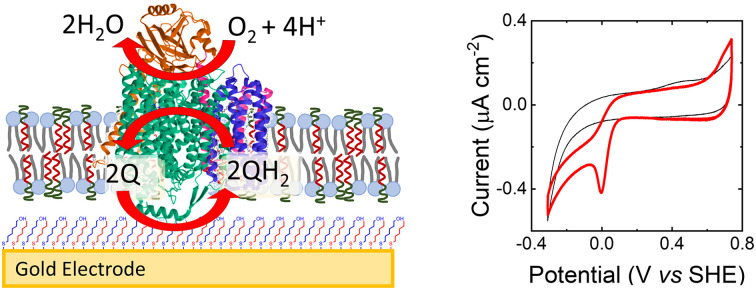

## Introduction

Hybrid polymer–lipid membranes consist of a blend of phospholipids and amphiphilic block copolymers. The resulting mixture exhibits synergistic properties that surpass those of the individual components.^1^ When in the form of hybrid vesicles (HVs), they combine the advantages of pure lipids (liposomes) and pure polymer (polymersomes) systems, offering biocompatibility to a diverse range of biomolecules along with enhanced stability and tuneable membrane properties.^2^ These combined properties make HVs highly versatile for applications such as drug delivery,^3^ biosensors^4^ and artificial cell development.^5^ HVs have also emerged as a promising platform for reconstituting membrane proteins.^2,6–8^

Solid-supported hybrid membranes (SSHMs) extend the concept of HVs to solid-supported systems, combining lipid bilayer properties with polymeric support.^9^ In bioelectrochemistry, solid-supported lipid membranes (SSLMs) have been widely used to study membrane redox enzymes,^10^ as they provide a functional lipid environment for electrochemical characterisation.^11^ However, their low mechanical stability limits their long-term applicability. In contrast, solid-supported polymer membranes (SSPLMs) offer greater membrane stability and can be chemically functionalised, enabling tailored surface properties for specific applications.^12^

So far, only a few studies have explored the properties of the hybrid membranes on solid supports and their combinations with membrane proteins. Indeed, their potential in fundamental studies of hybrid membranes and membrane proteins or applications in bioelectrocatalysis remains largely underexplored.

In 2014, Gettel *et al.* demonstrated that hybrid vesicles composed of POPC and PBd_22_–PEO_14_ can form homogeneous SSHM on solid surfaces through fusion, producing different structures depending on the surface energy: bilayers on hydrophilic surfaces, monolayers on hydrophobic surfaces, and mixed morphologies on amphiphilic surfaces.^13^ Further investigation in 2018 by Paxton *et al.* explored SSHMs formed using HVs composed of DOPC and PBd_37_–PEO_22_ on both planar and non-planar silica surfaces.^14^ They found that the hybrid bilayers maintained a continuous and homogeneous structure at lower polymer content. However, when the polymer content exceeded 50 mol%, the films became increasingly heterogeneous, showing a tendency toward vesicle adsorption rather than fusion. In 2020, Mumtaz Virk *et al.* similarly observed that HVs composed of POPC and PBd_22_-*b*-PEO_23_, with polymer content up to 50% w/w, formed smooth, defect-free planar surfaces on silica supports. However, similar weight fractions of DPPC lipids resulted in hybrid bilayers with nanoscale defects.^15^ Balestri *et al.* also confirmed micrometre-scale phase separation in SSHM composed of PBd_46_-*b*-PEO_30_ (from 10% to 65%) and DPPC onto silica substrates *via* spontaneous rupture and fusion of HVs.^16^ In 2021, Bello *et al.* investigated the behaviour of low molecular weight PBd_12_-*b*-PEO_9_ polymers mixed with POPC in supported hybrid bilayers.^17^ They found that stable bilayer formation and “lipid-like” properties were achieved with up to 50 mol% polymers, consistent with findings using higher molecular weight polymers. Vesicles with 50 mol% polymer content were more prone to deformation but effectively stabilized and homogenized the POPC distribution. Higher polymer concentrations resulted in phase separation. In 2024, Cardellini *et al.* found that gold nanoparticles (AuNP) preferentially clustered in the polymer-rich regions of the heterogeneous SSHM made of PBd_46_-*b*-PEO_34_ and DPPC.^18^ Recently, Schafer *et al.* demonstrated that mixing PBd_22_–PEO_14_ with POPC to form a SSHM enhances bilayer resilience, while maintaining biocompatibility, as evidenced by the successful incorporation of α-hemolysin.^19^ The Meier group studied SSHM monolayers and bilayers made from lipids (DPPC, DPPE, DOPC, POPE) and PDMS-*b*-PMOXA copolymers with varying PDMS lengths (16, 37, and 65 units).^20–22^ They found that phase separation and domain formation were most distinct with longer PDMS chains and saturated lipids, with the lipid headgroups affecting domain size and shape.^22^ By adjusting the polymer–lipid composition, they could control the insertion and distribution of the membrane protein MloK1. In these mixtures, membrane proteins preferentially inserted into the more fluid phase. Specifically, in mixtures with saturated lipids, the proteins localised in the polymer-rich phase, whereas with unsaturated lipids, they favoured the lipid-rich phase.

**Fig. 1:**
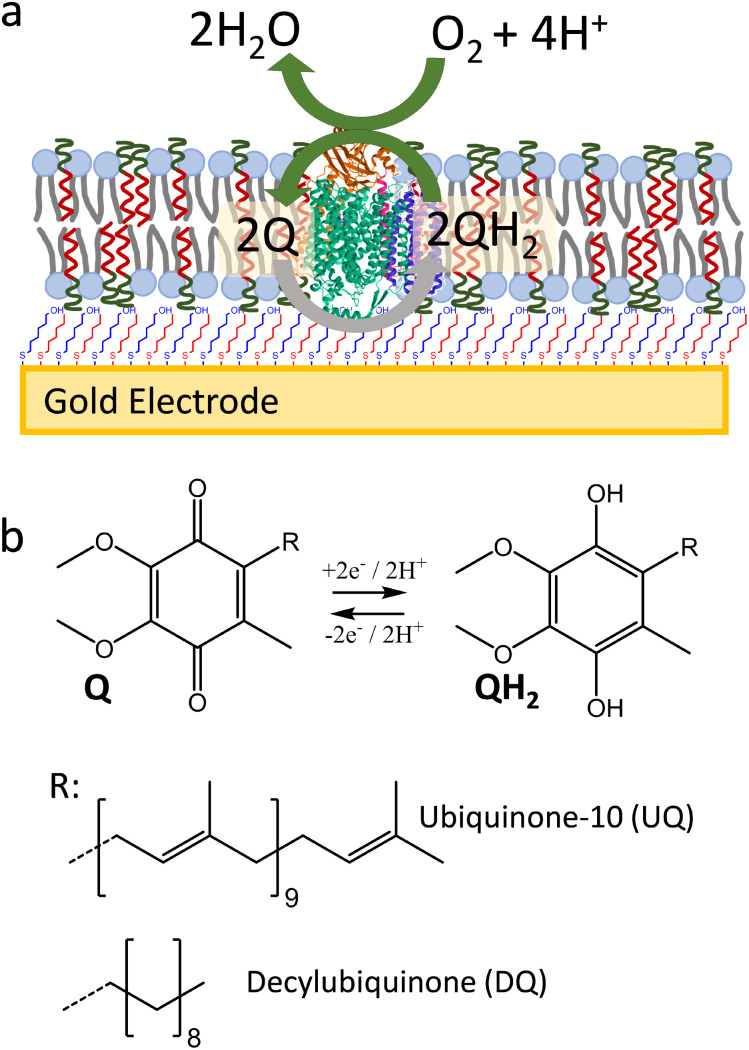
(a) Schematic representation of cyt *bo*_*3*_ in SSHM on gold electrode modified with a mixed SAM of MH : HT (1 : 1 molar ratio). In this system, ubiquinone is reduced at the electrode to ubiquinol, which is then reoxidized by cyt *bo*_*3*_, catalyzing oxygen reduction to water. (b) Structures of ubiquinone-10 (UQ) and decylubiquinone (DQ) in their fully oxidized (Q) and reduced forms (QH_2_).

Here, we show that SSHMs can be used as a platform to study the bioelectrocatalytic activity from a membrane redox enzyme, cytochrome *bo*_*3*_ (cyt *bo*_*3*_) from *Escherichia coli* (*E. coli*) (Fig. 1). Cyt *bo*_*3*_ is a four-subunit terminal oxidase complex (∼143 kDa) that belongs to the haem-copper oxidase enzyme family. It oxidises ubiquinol molecules while reducing molecular oxygen to water.^23^ The SSHM consisted of 1 : 1 molar ratio of poly(butadiene-*b*-ethylene oxide) (PBd_22_-*b*-PEO_14_; MW 1.8 kDa) amphiphilic block copolymer and *E. coli* polar lipid extracts. The SSHMs were formed onto a gold electrode coated with self-assembled monolayer (SAM) made of an optimised ratio of 6-mercapto-1-hexanol (MH) to hydrophobic 1-hexanethiol (HT) mixture (1 : 1 molar ratio). These hybrid bilayers were shown to efficiently cover the SAM-coated gold electrode, while allowing electrochemical oxidation and reduction of lipophilic quinones in the membranes. Cyt *bo*_*3*_ could be incorporated into the SSHM in an active form, as confirmed by its quinone : oxygen oxidoreductase activity.

## Experimental

### Materials

*E. coli* polar lipids were obtained from Avanti Polar Lipids (AL, U.S.A.). Block-copolymer poly(butadiene-*b*-ethylene oxide) (PBd_22_-*b*-PEO_14_, P9089–BdEO) was purchased from Polymer Source (Canada). If not otherwise stated, all the other chemicals were purchased from Merck. The compound mercapto-(ethylene-oxy)_3_-carbamate cholesterol (EO_3_C) was synthesized as previously described.^24^

### Cytochrome *bo*_*3*_ extraction and purification

Membrane protein cytochrome *bo*_*3*_ (cyt *bo*_*3*_) was expressed in *E. coli* GO105/pJRhisA as previously described.^25^ A freshly plated colony of *E. coli* GO105/pJRhisA was inoculated in LB containing 100 μg mL^−1^ carbenicillin and cultured at 37 °C at an agitation rate of 200 rpm for ∼16 h. This starter culture was then inoculated in LB medium (2% v/v) also supplemented with 100 μg mL^−1^ carbenicillin and 0.1 mM CuSO_4_. *E. coli* was grown to mid-logarithmic phase at 37 °C with shaking at 200 rpm for ∼6 h, until the optical density at 600 nm (OD_600nm_) reached 1.5. The cells were harvested by centrifugation at 7000*g* for 20 min at 4 °C and resuspended in W1 buffer (20 mM MOPS, 5 mM Mg_2_SO_4_, 30 mM Na_2_SO_4_) at a concentration of 0.25 g of wet cells per mL. *E. coli* cells suspension was passed twice through a cell disrupter (Constant Systems) at 30 kPsi. Cell debris was removed by centrifugation at 17 500*g* for 10 min at 4 °C. Cell membranes in the supernatant were then pelleted by ultracentrifugation at 200 000*g* for 90 min at 4 °C. To purify cyt *bo*_*3*_ in SMALPs, the membrane pellet was resuspended in 50 mM Tris–HCl (pH 8), 500 mM NaCl and 10% glycerol at a ‘wet weight’ concentration of 40 mg mL^−1^ (protein content ∼4 mg mL^−1^ as determine using a bicinchoninic acid assay (BCA) assay). Styrene maleic acid (SMA) copolymer (Cray Valley, SMA 2000 – MW 7.5 kDa) was added at a concentration of 2% w/v.^26^ The suspension was incubated for 2 h on a rotary shaker at RT and then centrifuged at 100 000*g* for 45 min at 4 °C to remove any non-solubilised proteins. SMA-solubilized proteins were incubated with pre-equilibrated Ni^2+^–NTA resin (Neo Biotech) for ∼16 h on a rotary shaker at 4 °C. The resin suspensions were loaded onto a gravity column, and the SMALP_cyt *bo*_*3*__ was eluted with 200 mM imidazole, 50 mM Tris–HCl (pH 8), 500 mM NaCl and 10% (vol/vol) glycerol. The imidazole was immediately removed after elution by performing 3 cycles of dilution in imidazole-free storage buffer (50 mM Tris and 150 mM NaCl, pH 8.0), and concentration using 100 kDa MW cut-off concentrator (VivaSpin). The purified SMALP_cyt *bo*_*3*__ were snap frozen in liquid nitrogen and stored at −20 °C until use. Protein concentration of purified cyt *bo*_*3*_ was determined *via* Soret Band at 409 nm (Nanodrop DeNovix DS-11) using extinction coefficient value *ε*_408 nm_ = 188 mM^−1^ cm^−1^.^27^

### Vesicles preparation

HVs were prepared using an adaptation of our previously described method.^28^*E. coli* polar lipid extracts (5 mg, 6.6 μmol) and PBd_22_-*b*-PEO_14_ (11.84 mg, 6.6 μmol) were each solubilised in chloroform, in 200 and 500 μL respectively, and mixed together in a glass vial. The organic solution was supplemented with decylubiquinone (DQ) (62.83 μg, 0.19 μmol, added as an aliquot taken from a stock solution 1 mg mL^−1^ in chloroform) or ubiquinol (UQ, 168.62 μg, 0.19 μmol, from a stock solution 1 mg mL^−1^ in chloroform). The mixture was then dried in a vacuumed desiccator for at least 2 h to form a thin lipid–copolymer. The lipid–copolymer films were resuspended in 1 mL of 20 mM 3-(*N*-morpholino)propanesulfonic acid (MOPS), 30 mM Na_2_SO_4_ (pH 7.4) to achieve a final concentration of 16.84 mg mL^−1^ (total lipid and copolymer mass). The suspension was incubated at 50 °C for 5 min and vortex for 1 min followed by five freeze–thaw–vortex cycles. The HVs were subsequently extruded 11 times through a 100 nm pore size polycarbonate membrane filter using an Avanti Mini-Extruder to form vesicles. Liposomes, made of *E. coli* lipids polar extract (Avanti Polar Lipids, AL, U.S.A.) were prepared by extrusion using similar methods at a final lipid concentration of 5 mg mL^−1^.

### Reconstitution of cyt *bo*_*3*_ into HVs

Incorporation of cyt *bo*_*3*_ into HVs was performed according to our previously published detergent-free reconstitution method.^29^ SMALP_cyt *bo*_*3*__ and HVs were incubated on ice for 30 min at a protein to lipid content ratio (w/w) of ∼1 : 100. MgCl_2_ was then added to a concentration of 10 mM and incubated with gentle shaking overnight at 4 °C. Mg^2+^ promotes SMA precipitation from solution forming a non-soluble complex that can be removed by centrifugation. The samples were subsequently centrifuged at 17 000*g* for 15 min to pellet SMA and non-reconstituted SMALP_cyt *bo*_*3*__. The supernatant, containing HVs with reconstituted cyt *bo*_*3*_, was collected and stored at 4 °C until use.

### Electrochemical measurements

Template stripped gold (TSG) and SAM surfaces were prepared as previously described.^30^ SAMs of mixed MH/EO_3_C (40 : 60) were prepared by incubation of TSG surface in 0.88 mM MH and 0.12 mM EO_3_C in propanol for 16 h. Mixed SAMs of MH/HT were formed by incubation of TSG surfaces with different molar ratios of 6-mercapto-1-hexanol (MH) and 1-hexanethiol (HT) to give a total of 1 mM thiol compounds in isopropanol (∼16 h). The SAM-coated TSG were rinsed with isopropanol and dried under nitrogen before being incorporated into a home-built electrochemical cell.^31^ Voltammetry and electrochemical impedance spectroscopy were carried out using an Autolab (Eco-chemie) electrochemical analyser equipped with a PGSTAT30 potentiostat, SCANGEN module and an FRA2 frequency analyser. All electrochemical measurements were performed in 2 mL 20 mM MOPS, 30 mM Na_2_SO_4_ buffer, pH 7.4. Electrochemical measurements were carried out in a three-electrode configuration electrochemical cell using mercury–mercury sulfate reference electrode and a Pt wire as counter electrode, as described previously.^31^ The electrochemical cell was housed in a Faraday cage and purged with nitrogen to remove oxygen. All reported potentials are quoted *versus* SHE (*E*_SHE_ = *E*_Hg_2_SO_4__ + 651 mV at 25 °C).

Solid-supported polymer–lipid hybrid membranes were formed adding liposomes or HVs into the electrochemical cell to final concentration of 0.5 mg mL^−1^ in 2 mL 20 mM MOPS, 30 mM Na_2_SO_4_ buffer supplemented with 10 mM CaCl_2_ at room temperature for 1 h. The HVs or liposome suspension was removed by rinsing the electrochemical cell three times with deionised water, three times with 1 mM ethylenediaminetetraacetic acid (EDTA) followed by 5 washes with 20 mM MOPS, 30 mM Na_2_SO_4_ buffer at pH 7.0.

### Quartz crystal microbalance with dissipation monitoring

QCM-D measurements were conducted on a Q-Sense E4 multifrequency QCM-D Instrument. Au crystals were cleaned by ultrasonication in 2% sodium dodecyl sulfate (SDS) detergent for 15 min, followed by rinsing and further ultrasonication in Milli-Q water for 15 min. The crystals were then dried under nitrogen and incubated in a UV-ozone (ultraviolet ozone cleaning system, low-pressure quartz-mercury vapor lamp emitting 254 and 185 nm UV, UVOCS) for 30 min. After UV-ozone treatment, the crystals were incubated in isopropanol for 30 min to reduce gold oxide after which the crystals then were transferred into the thiol solutions for 16 h followed by rinsing with isopropanol, as described under Electrochemical measurements. Prior to the measurements, the QCM-D chambers were filled with buffer to find the resonant frequencies of each overtone used. Experiments were performed at 22 °C at flow rate of 40 μL min^−1^ and a HV or liposome concentration of 0.5 mg mL^−1^ in 20 mM MOPS, 30 mM Na_2_SO_4_ pH 7.4. All data shown represent recordings at the seventh overtone.

### Atomic force microscopy sample preparation and imaging

MH : HT (50 : 50) SAM coated TSG electrodes, as described under electrochemical measurements, were glued to metal stubs using a layer of epoxy glue. AFM imaging was performed in liquid in PeakForce tapping mode using a Bruker Dimension FastScan Bio with Bruker ScanAsyst Fluid+ probes. Imaging was performed at room temperature in 20 mM MOPS, 30 mM Na_2_SO_4_ pH 7.4 buffer. HVs were incubated on the TSG electrodes at a concentration of 0.4 mg mL^−1^ in 10 mM CaCl_2_, 20 mM MOPS, 30 mM Na_2_SO_4_ pH 7.4 for 15 min. Excess vesicles were rinsed *via* fluid exchange using 5 exchanges of milli Q water followed by 5 exchanges of buffer whilst maintaining the membranes in a liquid environment.

## Results and discussion

### Solid-supported hybrid membranes (SSHMs)

Different methods have been developed to ‘support’ or couple lipid membranes to electrode surfaces for the study of redox-active membrane proteins.^32–35^ One common method is to ‘tether’ the membrane to the surface using lipid analogues that are chemically bonded to the electrode *via* a short linker, usually a poly–ethylene-glycol group. A second common approach is to ‘suspend’ the membrane above the electrode using non-covalent interactions. While various self-assembled monolayers (SAMs) on gold electrodes have been investigated for their ability to form such solid-supported lipid membranes (SSLMs), there is limited knowledge about which SAM is most suitable to form a solid-supported hybrid membrane (SSHM). Here, we tested both the tethered and suspended approach.

**Fig. 2:**
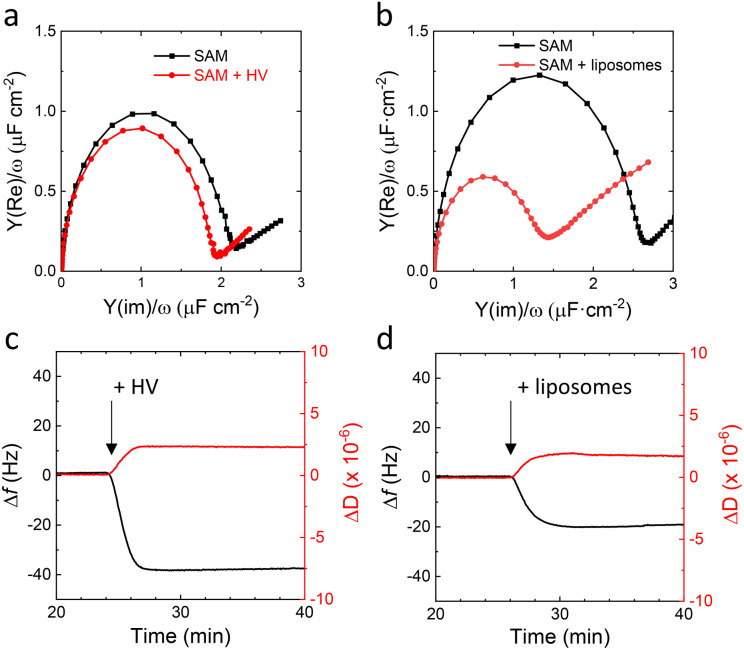
(a and b) Representative Cole–Cole plots of template-stripped gold with a mixed EO_3_C/MH (∼60 : 40) SAM before (black) and after (red) incubation with (a) HVs or (b) *E. coli* polar lipid extract liposomes (0.5 mg mL^−1^ of total PBd_22_-*b*-PEO_14_ polymer and lipid components, for 1 h at 20 °C, 10 mM CaCl_2_, 20 mM MOPS, 30 mM Na_2_SO_4_, pH 7.4). (c and d) QCM-D Δ*f* (black) and Δ*D* (red) shifts of gold sensors with mixed EO_3_C/MH (∼60 : 40) SAM upon incubation with either (c) HV or (d) *E. coli* polar lipid extract liposomes (0.5 mg mL^−1^ at 40 μL min^−1^, 22 °C, 20 mM MOPS, 30 mM Na_2_SO_4_, pH 7.4).

To test the tethered approach, a self-assembled monolayer (SAM) was prepared of a mixture of hydrophilic 6-mercapto-1-hexanol (MH) and the lipid ‘tether’, mercapto-(ethylene-oxy)_3_-carbamate cholesterol (EO_3_C) in a ∼40 : 60 ratio in the surface.^30^ We previously showed that EO_3_C-based SAMs are suitable to form well-defined tethered SSLMs, which can be used to electrochemically study cytochrome *bo*_*3*_ in *E. coli* lipid extracts.^25,31,36,37^ Well-formed tethered SSLMs on EO_3_C/MH surfaces are characterised by double layer capacitance of around 1 μF cm^−2^ by electrochemical impedance spectroscopy (EIS) and a frequency change of around −20 Hz with quartz crystal microbalance with dissipation (QCM-D) (Fig. 2b and d). Fig. 2b presents the EIS data in Cole–Cole plots, also known as normalised admittance plots, in which the diameter of the semicircle directly signifies the double layer capacitance, *C*_dl_. The −20 Hz frequency shift (Δ*f*) observed by QCM-D in Fig. 2d is slightly less than the ∼25 Hz typically observed for SSLM formation on SiO_2_ surfaces.^38^ This is because the EO_3_C ‘tether’ occupies part of the lower lipid leaflet in the tethered SSLM system.

In contrast to the tethered lipid systems, EIS and QCM-D indicate that HVs do not form impermeable planar SSHMs on EO_3_C-based SAMs (Fig. 2). After addition of HVs, no change in *C*_dl_ is observed with EIS and a higher Δ*f* (∼−40 Hz) is recorded by QCM-D (Fig. 2a and c). SSHMs are reported to have higher Δ*f* and Δ*D* values than those observed in SSLMs because membranes of HVs prepared with PBd_22_-*b*-PEO_14_ are thicker than lipid membranes and have different viscoelastic properties.^15^ The increase in viscoelasticity has been attributed to the extended poly(ethylene oxide) (PEO) chains interacting with the surrounding water and the more disordered hydrophobic core caused by the PBd chains.^39^ The QCM-D is thus not inconsistent with SSHM formation (see also below), but the EIS data indicate that the HVs are unable to form an impermeable SSHM on the surface. We hypothesize this is due to the length of the hydrophilic PEO chain of PBd_22_-*b*-PEO_14_, which is mismatched in size with the relatively short linker of the EO_3_C tether.

**Fig. 3:**
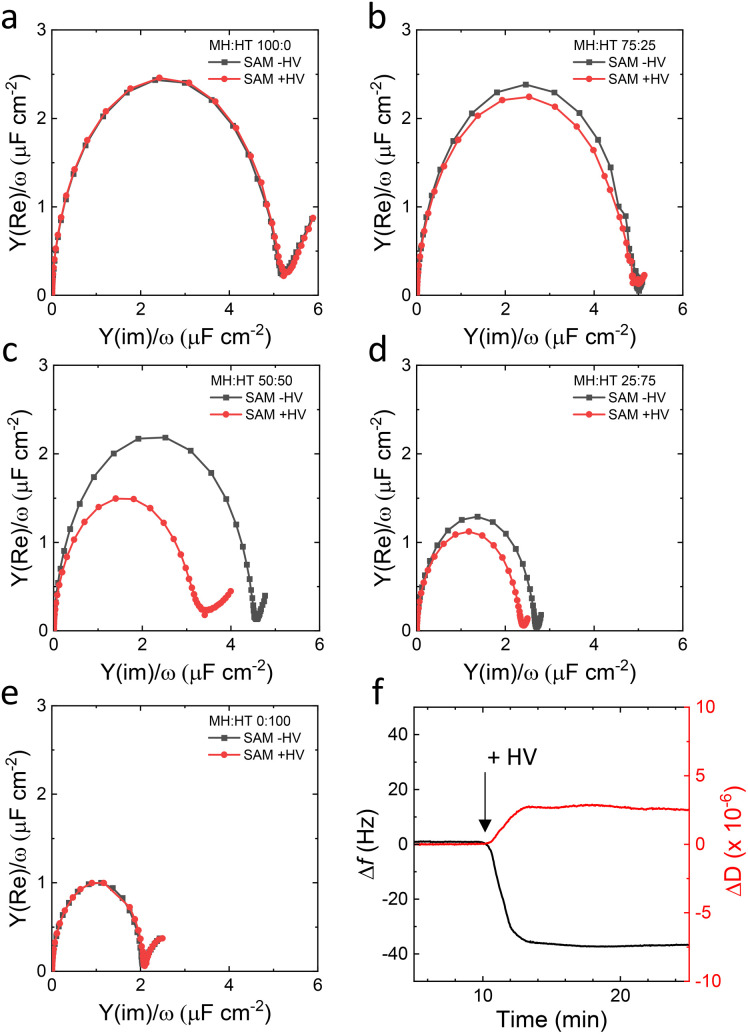
(a–e) Representative Cole–Cole plots of self-assembled monolayers on template-stripped gold with different ratio of the mixture 6-mercapto-1-hexanol (MH) : 1-hexanethiol (HT) (a: 100 : 0, b: 75 : 25, c: 50 : 50, d: 25 : 75 and e: 0 : 100), (black) before and (red) after incubation with HVs (0.5 mg mL^−1^ for 1 h at 20 °C, 10 mM CaCl_2_, 20 mM MOPS, 30 mM Na_2_SO_4_). (f) QCM-D Δ*f* (black) and Δ*D* (red) shifts of gold sensors with mixed MH : HT (50 : 50) SAM upon addition of 0.5 mg mL^−1^ per HV (40 μL min^−1^, 22 °C, 20 mM MOPS, 30 mM Na_2_SO_4_, pH 7.4).

As the tethered systems could not form impermeable SSHMs, a non-tethered approach was considered. We chose to investigate the formation of SSHM on mixed SAMs of hydrophilic 6-mercapto-1-hexanol (MH) and hydrophobic 1-hexanethiol (HT). Fig. 3a–e (black traces) show EIS results of SAMs prepared from different stoichiometric ratios of MH and HT in solution (MH : HT 100 : 0, 75 : 25, 50 : 50, 25 : 75, 0 : 100). It is known that *C*_dl_ differs greatly between hydrophobic and hydrophilic surfaces^40^ and the *C*_dl_ values obtained here for pure MH and HT SAM (5 μF cm^−2^ and 2 μF cm^−2^, respectively) are in line with previous values reported by us and others.^30,41^ Notably, *C*_dl_ decreases with an increase in the fraction of the hydrophobic HT in the SAM, indicating that mixed monolayers are formed (Fig. 3a–e, black traces). Elucidating why *C*_dl_ decreases non-linearly with the MH : HT ratio is beyond the remit of this study. Fig. 3a–e also include the EIS results of MH : HT surfaces of different ratios after incubation with HVs (red symbols and lines). For all SAMs except for the 50 : 50 ratio, following deposition of the hybrid membrane, no or marginal reductions in *C*_dl_ are observed. This implies that an incomplete or no SSHM forms on SAM compositions of MH : HT with 100 : 0, 75 : 25, 27 : 75 and 0 : 100 ratio (see also Fig. S1†). In contrast, for the 50 : 50 ratio, a significant reduction in *C*_dl_ is observed (Fig. 3c), which is indicative of SSHM formation. *C*_dl_ of the 50 : 50 MH : HT SAM, as determined from the diameter of the semi-circle in the Cole–Cole plot, is measured to be 4.17 ± 0.13 μF cm^−2^, decreasing to 2.80 ± 0.08 μF cm^−2^ after incubation with HVs. Interestingly, the same 50 : 50 MH : TH SAM interface does not support the formation of impermeable planar SSLM by liposomes (Fig. S2†). Upon liposome addition, EIS shows no noticeable change in *C*_dl_ (Fig. S2a†), and QCM-D instead detects a substantial frequency shift (∼−175 Hz), indicating vesicle adsorption rather than fusion into a continuous bilayer (Fig. S2b†).

To assess the role of polymer content, we attempted to form SSHMs using HVs with a PBd_22_-*b*-PEO_14_ polymer molar ratio of 25% on both EO_3_C : MH and MH : HT SAMs. In both cases, no significant reduction in *C*_dl_ was observed, indicating that this composition does not effectively support SSHM formation (Fig. S3†). Higher polymer content (≥75%) was not investigated, as our previous work has shown that cyt *bo_3_* exhibits little activity in such compositions.^28^

Confirmation of SSHM formation on 50 : 50 MH : HT electrodes was sought by QCM-D and atomic force microscopy (AFM). In Fig. 3f, the addition of HV (50% PBd_22_-*b*-PEO_14_) to the MH : HT (50 : 50) SAM coated QCM-D sensors is consistent with SSHM formation: a sharp decrease is followed by an almost constant frequency with Δ*f* = −37 Hz. Similarly, in the dissipation profiles, a sharp increase in dissipation, followed by an almost constant value, is observed, with Δ*D* of 2.5 × 10^−6^. These profiles and changes in Δ*f* and Δ*D* agree with previous similar reports on SSHM formation.^18,42^

**Fig. 4:**
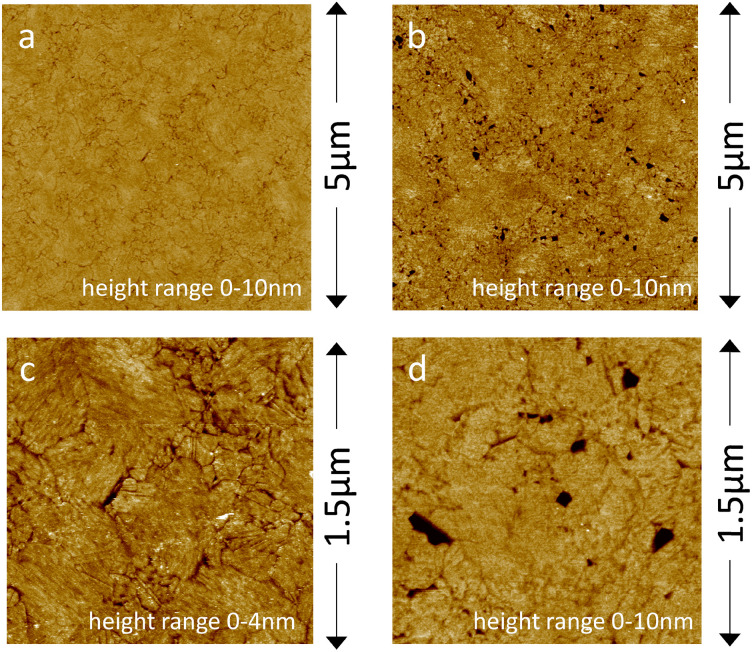
AFM images of template-stripped gold surfaces with a MH : HT (50 : 50) SAM (a and c) before and (b and d) after the formation of SSHM (0.5 mg mL^−1^ per HV for 1 h at 20 °C, 10 mM CaCl_2_, 20 mM MOPS, 30 mM Na_2_SO_4_, pH 7.4).

Fig. 4a and c show the planar and homogenous surface morphology of the MH : HT (50 : 50) SAM coated electrodes as studied with AFM. AFM confirms that upon addition of HVs, a planar SSHM is formed (Fig. 4b and d). The SSHM displays a planar and uniform surface structure with some defects. No visible signs of phase separation were observed, unlike hybrid membranes based on different copolymers.^20,21^ We have previously demonstrated that HVs made of PBd_22_-*b*-PEO_14_ exhibit homogeneous membrane structures without evidence of phase-separated domains.^43,44^

### Electrochemical study of cytochrome *bo*_*3*_ in SSHM

The activity of membrane proteins in SSHM was assessed using the model enzyme, cytochrome *bo*_*3*_ (cyt *bo*_*3*_), which is an *E. coli* ubiquinol : oxygen oxidoreductase. We have previously shown that the activity of cyt *bo*_*3*_ can be studied by the electrochemical reduction of the lipophilic ubiquinone-10 substrate in the SSLM.^25,31,36,37^ As the electrochemically formed ubiquinol-10 is catalytically re-oxidised to ubiquinone-10 by cyt *bo*_*3*_, cyclic voltammetry shows a characteristic catalytic wave, confirming cyt *bo*_*3*_ activity.

**Fig. 5:**
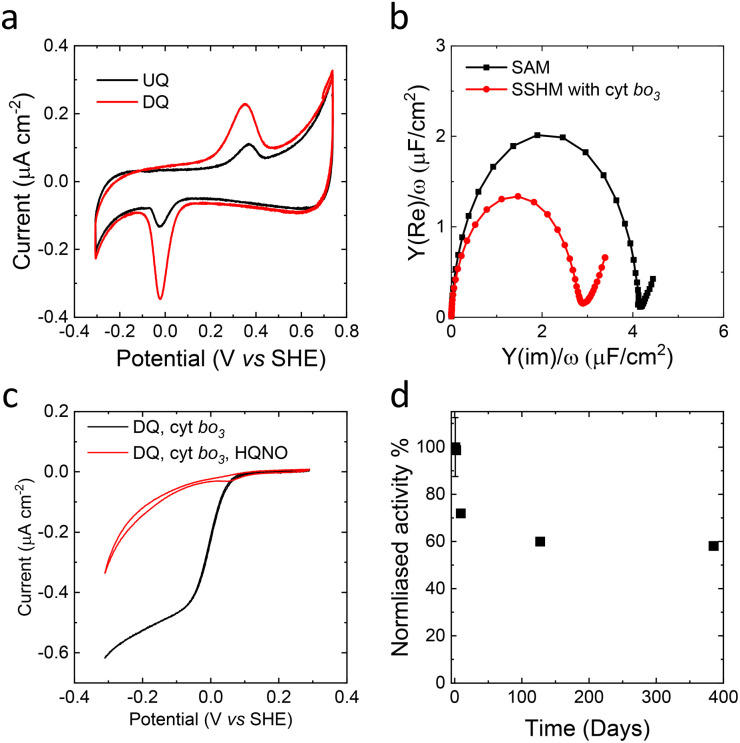
(a) Cyclic voltammograms (10 mV s^−1^) of a SSHM on MT : HT (50 : 50). The SSHM is prepared with 1.5% molar ratio (quinone to polymer + lipid) of UQ (black) or decylubiquinone (DQ) (red). (b) Representative Cole–Cole plots of mixed self-assembled monolayers (MH : HT, 50 : 50) on template-stripped gold (black) before and (red) after incubation with HV containing cyt *bo*_*3*_. (c) Cyclic voltammograms (1 mV s^−1^) of cyt *bo*_*3*_ containing SSHM with 1.5% molar ratio DQ (black) before and (after) after addition of the inhibitor 100 μM HQNO. (d) Normalized activity of cyt *bo*_*3*_ in a SSHM after storing the HV/cyt *bo*_*3*_ at 4 °C at the indicated period (independent samples). Due to the length of time, this experiment was only performed once for samples that were stored longer than 1 week.

We investigated the electrochemical behaviour of SSHM (on SAMs of MH : HT, 50 : 50) enriched with ubiquinone-10 (UQ) and decylubiquinone (DQ) using cyclic voltammetry (CV) (Fig. 5a). Comparison of peak area of the redox peaks of UQ and DQ shows much higher electroactive coverage of DQ compared to UQ, even though their concentrations in the SSHM are approximately the same (Fig. 5a). We hypothesize that the PEG ‘layer’ in the SSHM, which is formed by PBd_22_-*b*-PEO_14_ at the interface between SAM and the planer membrane, might increase the distance between the quinone headgroup and the electrode surface. It is possible that DQ, which is less hydrophobic than UQ (Fig. 1b), more readily diffuses into the PEG layer and therefore shows improved electrochemical behaviour. As DQ displayed better electrochemical performance compared to UQ, we used this quinone to study the activity of cyt *bo*_*3*_.

Cyt *bo*_*3*_ was purified from *E. coli* in styrene maleic acid lipid particles (SMALPs) and reconstituted in HVs using our recently developed detergent-free reconstitution method.^29^ The cyt *bo*_*3*_-containing HVs were subsequently used to form the SSHM on MH : HT SAMs. Comparison of EIS of SSHM from HV with and without cyt *bo*_*3*_ (Fig. 5b) shows that the inclusion of cyt *bo*_*3*_ has almost no effect on the *C*_dl_ of the formed SSHM, suggesting that cyt *bo*_*3*_ does not majorly affect the permeability or structure of the SSHM. Cyclic voltammetry (CV) of the cyt *bo*_*3*_-containing SSHM shows the expected catalytic wave, with catalytic currents in the same order of magnitude as those previously obtained on the ‘tethered’ SSLM with similar amounts of cyt *bo*_*3*_.^37^ Inhibition with HQNO confirms the activity is due to cyt *bo*_*3*_ (Fig. 5c). We estimated the surface coverage of cyt *bo*_*3*_ in SSHMs using two independent approaches. First, we calculated the enzyme density based on the surface area and mass composition of the membrane. The membranes were prepared with a 1 : 1 molar ratio of *E. coli* lipids and PBd_22_–PEO_14_, with molecular areas of 0.61 nm^2^ for POPE, as *E. coli* lipids are ∼80% POPE,^45^ and 0.63 nm^2^ for PBd–PEO.^39^ Given that cyt *bo*_*3*_ accounts for 0.74 mass percent of the total membrane mass,^29^ this analysis yields an estimated coverage of 18 fmol cm^−2^ (ranging from 14–21 fmol cm^−2^).

Second, we estimated the enzyme density from the catalytic activity on the electrode (∼450 nA cm^−2^, Fig. 5c) and the known maximum activity of cyt *bo*_*3*_ in solution (540 e^−^ per s).^46^ This calculation gives an enzyme coverage of 8.6 fmol cm^−2^.

Both estimates are in good agreement with our previous findings for cyt *bo*_*3*_ (ref. 37 and 47) in SSLMs and provide confidence that ∼50% of the incorporated cyt *bo_3_* remains electrochemically active in SSHMs.

Our SSHM approach overcomes the limitations observed in fully polymer-based solid supported membrane for membrane protein. Previous studies on PBd–PEO solid-supported membranes reported a fivefold lower reconstitution efficiency for αHL.^48^ This highlights the advantage of hybrid lipid–polymer systems in balancing membrane stability with protein functionality, overcoming the reduced incorporation typically seen in polymer-only platforms.

We have previously shown that cyt *bo*_*3*_ displays a greatly increased durability at 4 °C, when reconstituted in HV compared to reconstitution in liposomes.^28,49^ Here, we confirmed that cyt *bo*_*3*_ in HV stored at 4 °C for over one year can still form SSHM with a similar EIS profile to a freshly prepared sample, and retains over 50% of its original activity (Fig. 5d and S4†). We note, however, that when monitoring the activity of cyt *bo*_*3*_ on the surface within SSHM, all activity is lost after storing the SSHM for >16 h at 4 °C, although the *C*_dl_ remains relatively unchanged (Fig. S5a†). Notably, the quinone peak, which is critical for the catalytic activity of cyt *bo*_*3*_, is diminished after overnight storage as SSHM (Fig. S5b†). This reduction may indicate a loss of redox mediators from the SSHM, contributing significantly to the observed loss of activity. Indeed, we observed that quinone depletion occurs in SSHMs even without cyt *bo_3_* (Fig. S5d†), while *C*_dl_ remains nearly unchanged (Fig. S5c†), indicating that the membrane remains stable. Attempts to replenish quinones within the membrane by adding additional quinones to the bulk media above did not recover the lost function. As some quinone molecules remain while cyt *bo_3_* activity is entirely lost, quinone depletion alone does not fully explain the loss of enzymatic function. We therefore hypothesize that, although the HV stabilizes cyt *bo*_*3*_, it is unable to compensate for the destabilizing effect that a gold/SAM interface might impose on proteins. Hence, there is significant scope for the future engineering of the hard-soft, electrode–membrane interface of SSHMs that can retain the longevity of membrane enzyme activity, which the hybrid membrane environment is capable of providing.

## Conclusions

We have demonstrated that hybrid vesicles prepared from PBd_22_-*b*-PEO_14_ and *E. coli* lipids extract can form planar solid-supported hybrid membranes (SSHM) on mixed self-assembled monolayers from 6-mercapto-1-hexanol and 1-hexanethiol (50 : 50 ratio). Membrane proteins in SSHM remain active as shown by the redox activity of the membrane enzyme cyt *bo*_*3*_, which was detected electrochemically using lipophilic quinone substrates as redox mediator.

Importantly, our findings highlight the non-interchangeability of SAMs between lipid and polymer–lipid hybrid membranes. While the MH : HT SAM at a 50 : 50 ratio was highly effective for forming homogenous and impermeable SSHMs with HVs, it did not support the formation of solid-supported lipid membranes (SSLM) from liposomes. In contrast, SAMs optimized for liposomes, such as those containing mercapto-(ethylene-oxy)_3_-carbamate cholesterol (EO_3_C), failed to form impermeable SSHMs with HVs. This shows the need to tailor surface chemistries specifically for each membrane system. Building on this, further investigations into SAMs formed from thiols with varying chain lengths or headgroup functionalities could provide additional insights into expanding SSHM applicability across different membrane architectures.

Our study also provides new insights into the stability and longevity of cyt *bo*_*3*_ reconstituted in HVs. We found that HV with cyt *bo*_*3*_ maintain their capability to form SSHMs with active enzyme even after one year of storage at 4 °C. However, despite this enhanced stability, a complete loss of activity was observed when SSHMs containing cyt *bo*_*3*_ were stored for more than 16 hours at 4 °C. Looking forward, there is significant potential to improve SSHM designs to better preserve membrane enzyme activity. The potential tunability of the physical and chemical properties of SSHMs opens new avenues to study complex cellular processes and broaden the applications of hybrid membranes in biosensing and bioelectrocatalysis.

## Data availability

The data files supporting figures in the manuscript and the ESI† are available in the Research Data Leeds repository at https://doi.org/10.5518/1592.

## Author contributions

R. C., designed and performed all experiments and analysed the resulting data. G. R. H. performed the AFM experiments and analysed the resulting data. M. R., S. P. M., P. A. B. and L. J. C. J. conceived the project. P. A. B. and L. J. C. J. directed the work. R. C., P. A. B. and L. J. C. J. wrote the initial manuscript. All authors have contributed and given approval to the final version of the manuscript.

## Conflicts of interest

The authors declare no competing financial interest.

## Supplementary Material

LF-002-D4LF00362D-s001

## References

[cit1] Go Y. K., Leal C. (2021). Chem. Rev..

[cit2] Beales P. A., Khan S., Muench S. P., Jeuken L. J. (2017). Biochem. Soc. Trans..

[cit3] Reimhult E., Virk M. M. (2021). J. Biomed. Res..

[cit4] Wang L., Zeng X., Shen W., Tang S., Lee H. K. (2023). TrAC, Trends Anal. Chem..

[cit5] Lu Y., Allegri G., Huskens J. (2022). Mater. Horiz..

[cit6] Brodszkij E., Städler B. (2024). Chem. Sci..

[cit7] Palivan C. G., Goers R., Najer A., Zhang X., Car A., Meier W. (2016). Chem. Soc. Rev..

[cit8] Shin J., Cole B. D., Shan T., Jang Y. (2022). Biomacromolecules.

[cit9] Eggenberger O. M., Jaśko P., Tarvirdipour S., Schoenenberger C.-A., Palivan C. G. (2023). Helv. Chim. Acta.

[cit10] Küchler A., Yoshimoto M., Luginbühl S., Mavelli F., Walde P. (2016). Nat. Nanotechnol..

[cit11] Castellana E. T., Cremer P. S. (2006). Surf. Sci. Rep..

[cit12] Belegrinou S., Menon S., Dobrunz D., Meier W. (2011). Soft Matter.

[cit13] Gettel D. L., Sanborn J., Patel M. A., de Hoog H.-P., Liedberg B., Nallani M., Parikh A. N. (2014). J. Am. Chem. Soc..

[cit14] Paxton W. F., McAninch P. T., Shin S. H. R., Brumbach M. T. (2018). Soft Matter.

[cit15] Mumtaz Virk M., Hofmann B., Reimhult E. (2019). Langmuir.

[cit16] Balestri A., Chiappisi L., Montis C., Micciulla S., Lonetti B., Berti D. (2020). Langmuir.

[cit17] Bello G., Cavallini F., Dailey L. A., Ehmoser E.-K. (2021). Biochim. Biophys. Acta, Biomembr..

[cit18] Cardellini J., Balestri A., Comparini L., Lonetti B., Brucale M., Valle F., Berti D., Montis C. (2024). J. Colloid Interface Sci..

[cit19] Schafer E. A., Davis E., Manzer Z., Daniel S., Rivnay J. (2023). ACS Appl. Mater. Interfaces.

[cit20] Di Leone S., Avsar S. Y., Belluati A., Wehr R., Palivan C. G., Meier W. (2020). J. Phys. Chem. B.

[cit21] Di Leone S., Kyropoulou M., Köchlin J., Wehr R., Meier W. P., Palivan C. G. (2022). Langmuir.

[cit22] Kowal J., Wu D., Mikhalevich V., Palivan C. G., Meier W. (2015). Langmuir.

[cit23] García-Horsman J. A., Barquera B., Rumbley J., Ma J., Gennis R. B. (1994). J. Bacteriol..

[cit24] Boden N., Bushby R. J., Liu Q., Evans S. D., Jenkins A. T. A., Knowles P. F., Miles R. E. (1998). Tetrahedron.

[cit25] Weiss S. A., Bushby R. J., Evans S. D., Jeuken L. J. C. (2010). Biochim. Biophys. Acta, Bioenerg..

[cit26] Lee S. C., Knowles T. J., Postis V. L. G., Jamshad M., Parslow R. A., Lin Y.-P., Goldman A., Sridhar P., Overduin M., Muench S. P., Dafforn T. R. (2016). Nat. Protoc..

[cit27] Osborne J. P., Cosper N. J., Stälhandske C. M. V., Scott R. A., Alben J. O., Gennis R. B. (1999). Biochemistry.

[cit28] Khan S., Li M., Muench S. P., Jeuken L. J., Beales P. A. (2016). Chem. Commun..

[cit29] Catania R., Machin J., Rappolt M., Muench S. P., Beales P. A., Jeuken L. J. C. (2022). Macromolecules.

[cit30] Jeuken L. J. C., Daskalakis N. N., Han X., Sheikh K., Erbe A., Bushby R. J., Evans S. D. (2007). Sens. Actuators, B.

[cit31] Weiss S. A., Bushby R. J., Evans S. D., Henderson P. J. F., Jeuken L. J. C. (2009). Biochem. J..

[cit32] Alvarez-Malmagro J., García-Molina G., López De Lacey A. (2020). Sensors.

[cit33] Jeuken L. J. C. (2009). Nat. Prod. Rep..

[cit34] Su Z., Leitch J. J., Lipkowski J. (2018). Curr. Opin. Electrochem..

[cit35] Zhang H., Catania R., Jeuken L. J. C. (2020). Catalysts.

[cit36] Heath G. R., Li M., Rong H., Radu V., Frielingsdorf S., Lenz O., Butt J. N., Jeuken L. J. C. (2017). Adv. Funct. Mater..

[cit37] Jeuken L. J. C., Connell S. D., Henderson P. J. F., Gennis R. B., Evans S. D., Bushby R. J. (2006). J. Am. Chem. Soc..

[cit38] Richter R. P., Berat R., Brisson A. R. (2006). Langmuir.

[cit39] Müller W. A., Beales P. A., Muniz A. R., Jeuken L. J. C. (2023). Biomacromolecules.

[cit40] Sur U. K., Lakshminarayanan V. (2002). J. Colloid Interface Sci..

[cit41] Gaigalas A. K., Niaura G. (1997). J. Colloid Interface Sci..

[cit42] Willes K. L., Genchev J. R., Paxton W. F. (2020). Polymer.

[cit43] Seneviratne R., Catania R., Rappolt M., Jeuken L. J. C., Beales P. A. (2022). Soft
Matter.

[cit44] Seneviratne R., Coates G., Xu Z., Cornell C. E., Thompson R. F., Sadeghpour A., Maskell D. P., Jeuken L. J. C., Rappolt M., Beales P. A. (2023). Small.

[cit45] Murzyn K., Róg T., Pasenkiewicz-Gierula M. (2005). Biophys. J..

[cit46] Rumbley J. N., Nickels E. F., Gennis R. B. (1997). Biochim. Biophys. Acta, Protein Struct. Mol. Enzymol..

[cit47] Jeuken L. J. C., Weiss S. A., Henderson P. J. F., Evans S. D., Bushby R. J. (2008). Anal. Chem..

[cit48] Zhang X., Fu W., Palivan C. G., Meier W. (2013). Sci. Rep..

[cit49] Seneviratne R., Khan S., Moscrop E., Rappolt M., Muench S. P., Jeuken L. J. C., Beales P. A. (2018). Methods.

