# Supplementary material for: Solid-supported polymer–lipid hybrid membrane for bioelectrochemistry of a membrane redox enzyme
Source: RSC Appl Interfaces. 2025 Feb 11;2(3):665–72. doi: 10.1039/d4lf00362d (PMC11834424; doi:10.1039/d4lf00362d)
Supplement: LF-002-D4LF00362D-s001 [file LF-002-D4LF00362D-s001.pdf]

Supplementary Information (SI) for RSC Applied Interfaces.
This journal is © The Royal Society of Chemistry 2025

Supporting Information:

Solid-supported polymer-lipid hybrid membrane for bioelectrochemistry of a membrane redox enzyme

Rosa Catania,[ab] George R. Heath,[bc] Michael Rappolt,[d] Stephen P. Muench,[be] Paul A. Beales[ab] and Lars J.C. Jeuken*[f]*

[a] School of Chemistry, University of Leeds, Leeds LS2 9JT (UK)

[b] Astbury Centre for Structural Molecular Biology, University of Leeds, Leeds LS2 9JT (UK)

[c] School of Physics and Astronomy, University of Leeds, Leeds LS2 9JT (UK)

[d] School of Food Science and Nutrition, University of Leeds, Leeds LS2 9JT (UK)

[e] School of Biomedical Sciences, Faculty of Biological Sciences, University of Leeds, Leeds LS2 9JT (UK)

[f] Leiden Institute of Chemistry, Leiden University, PO Box 9502, 2300 RA, Leiden, the Netherlands.

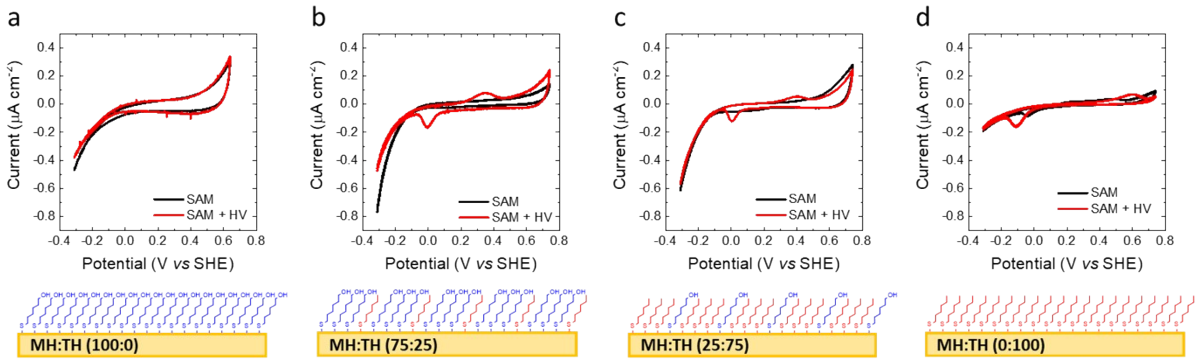

Fig. S1 Representative cyclic voltammetry (CV) of solid-supported polymer-lipid hybrid membrane (SSHM) on template-stripped gold with different ratio of the mixture 6-mercapto-1-hexanol (MH):1-hexanethiol (HT) at different ratios (a: 100:0, b: 75:25, c: 25:75 and d: 0:100), (black) before and (red) after incubation with HV (0.5 mg/mL for 1 h at 20°C, 10 mM $CaCl_2$, 20 mM MOPS, 30 mM Na_2SO_4) containing 1.5% molar ratio DQ.

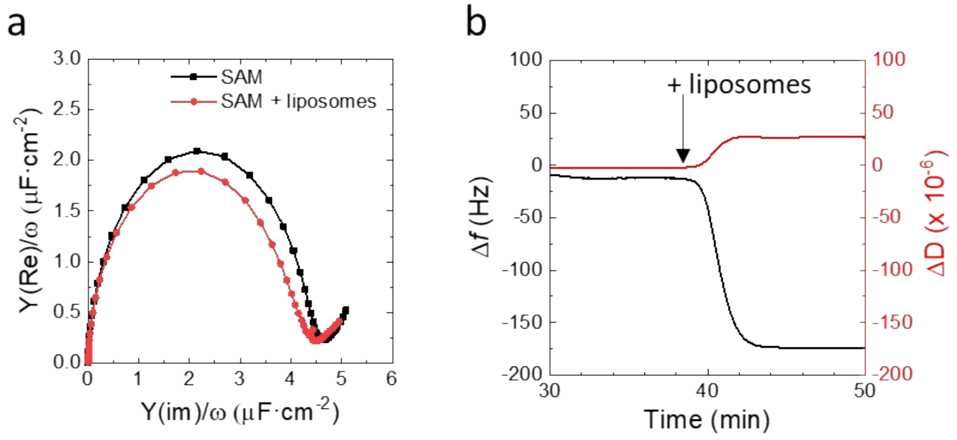

Fig. S2 (a) Representative Cole–Cole plot of mixed self-assembled monolayers (MH:HT, 50:50) on template-stripped gold (black) before and (red) after incubation with *E. coli* polar lipid extract liposomes (0.5 mg/mL, for 1 h at 20°C, 10 mM $CaCl_2$, 20 mM MOPS, 30 mM Na_2SO_4, pH 7.4). (b) QCM-D Δf (black) and ΔD (red) shifts of gold sensors with mixed MH:HT (50:50) SAM upon addition of *E. coli* polar lipid extract liposomes (0.5 mg/mL at 40 µL/min, 22°C, 20 mM MOPS, 30 mM Na_2SO_4, pH 7.4).

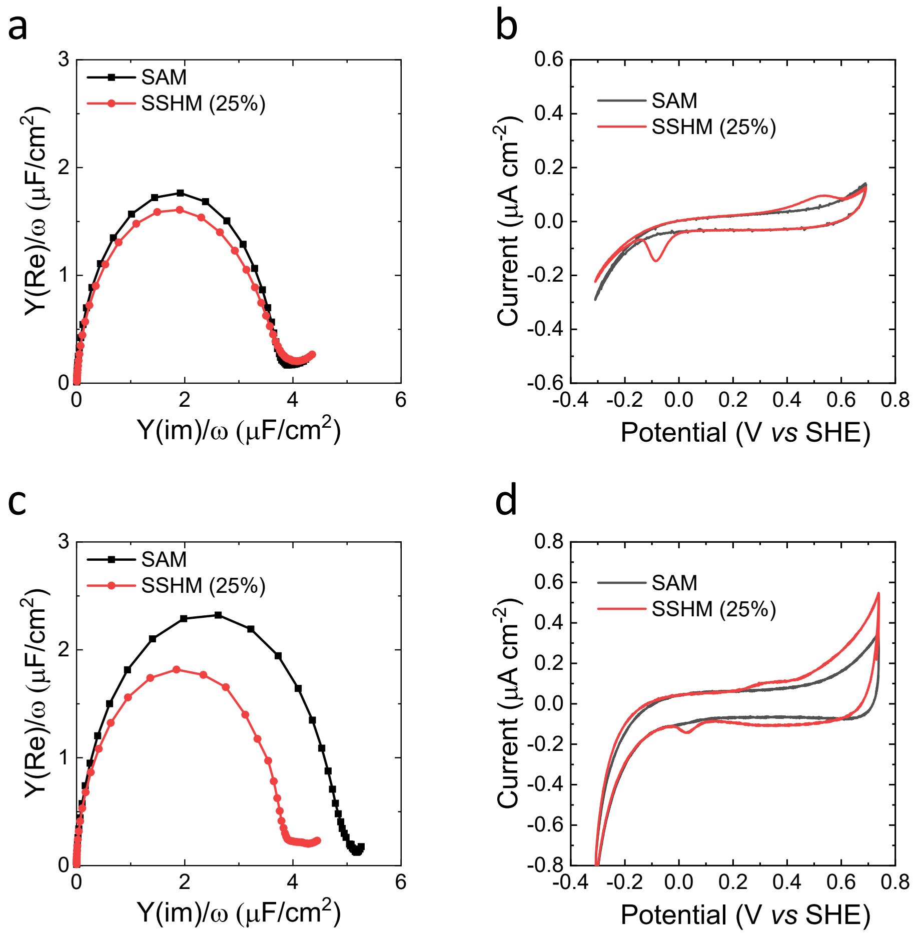

Fig. S3 Cole–Cole plots and cyclic voltammograms (10 mV/s) of SSHM prepared from HVs with a polymer molar ratio of 25% on EO_3C/MH (~60:40) SAM (a, b) and on MT:HT (50:50) SAM (c, d). The data shown represent before (black) and after (red) incubation with HVs (0.5 mg/mL of total PBd_{22}-b-PEO_{14} polymer and lipid components, for 1 h at 20 °C, 10 mM $CaCl_2$, 20 mM MOPS, 30 mM Na_2SO_4, pH 7.4).

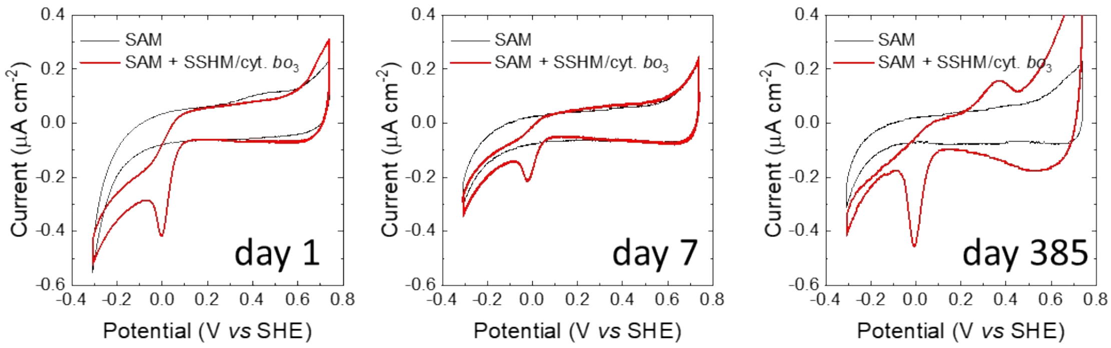

Fig. S4 Cyclic voltammetry of SSMH prepared from cyt bo_3/HV samples that are 1, 7 and 385 days old, as indicated.

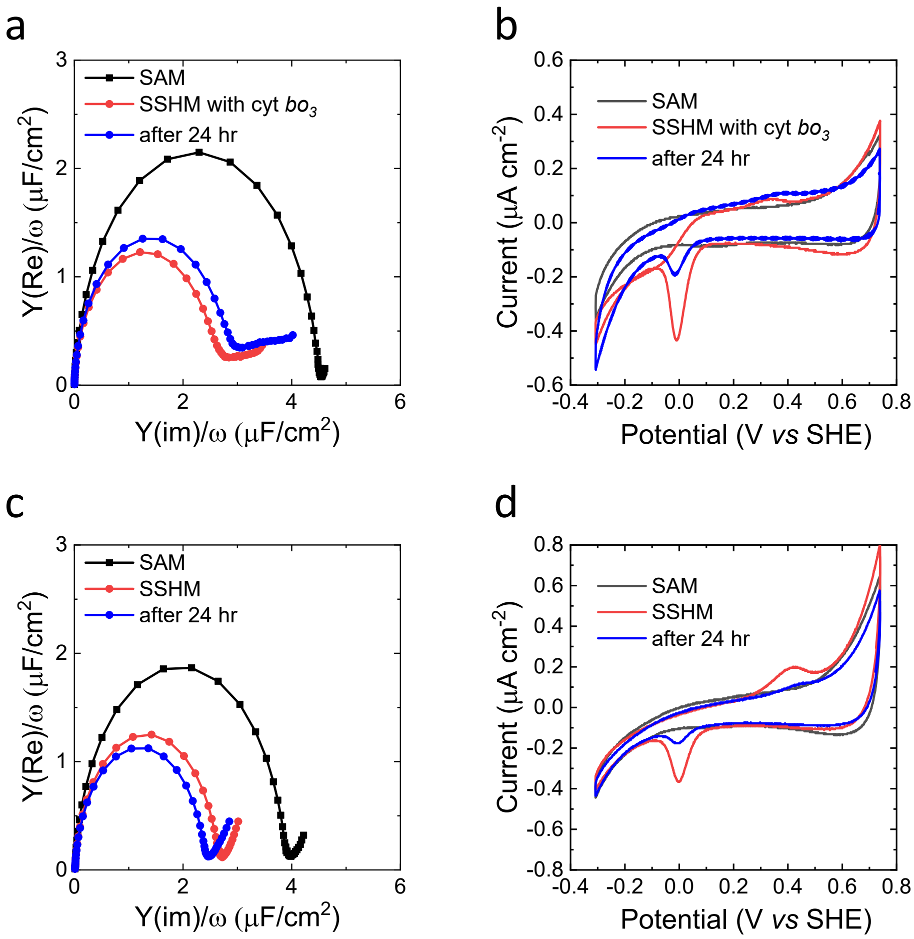

Fig. S5 Cole–Cole plots and cyclic voltammograms (10 mV/s) of SSHM prepared from HV with (a and b) and without (c and d) cyt bo_3 on MT:HT (50:50). The data shown represent before (black) and after (red) incubation with HVs, and after 24 hours (blue) of storage as SSHM at 4°C.
